# A Low Input Current and Wide Conversion Ratio Buck Regulator with 75% Efficiency for High-Voltage Triboelectric Nanogenerators

**DOI:** 10.1038/srep19246

**Published:** 2016-01-19

**Authors:** Li-Chuan Luo, De-Chun Bao, Wu-Qi Yu, Zhao-Hua Zhang, Tian-Ling Ren

**Affiliations:** 1 Beijing Institute of Nanoenergy and Nanosystems, Chinese Academy of Sciences, Beijing 100083, China; 2 Institute of Microelectronics & Tsinghua National Laboratory for Information Science and Technology (TNLIST), Tsinghua University, Beijing 100084, China

## Abstract

It is meaningful to research the Triboelectric Nanogenerators (TENG), which can create electricity anywhere and anytime. There are many researches on the structures and materials of TENG to explain the phenomenon that the maximum voltage is stable and the current is increasing. The output voltage of the TENG is high about 180–400 V, and the output current is small about 39 μA, which the electronic devices directly integration of TENG with Li-ion batteries will result in huge energy loss due to the ultrahigh TENG impedance. A novel interface circuit with the high-voltage buck regulator for TENG is introduced firstly in this paper. The interface circuit can transfer the output signal of the TENG into the signal fit to a lithium ion battery. Through the circuit of the buck regulator, the average output voltage is about 4.0 V and the average output current is about 1.12 mA. Further, the reliability and availability for the lithium ion battery and the circuit are discussed. The interface circuit is simulated using the Cadence software and verified through PCB experiment. The buck regulator can achieve 75% efficiency for the High-Voltage TENG. This will lead to a research hot and industrialization applications.

There are many researches to put the electrostatic energy as a part of the self-powered portable electronics in the recent years^1^. With the tremendous increase number of mini electronics, it is vitally important to develop energy storage related technology. This is the field of nano-energy, which is the power for sustainable, maintenance free and self-powered operation of nano-systems^2^,^3^. Recently, Professor Wang invented the triboelectric nano-generator (TENG), which is used to convert mechanical energy into electricity by a conjunction of triboelectrification and electrostatic induction^4^,^5^,^6^,^7^. However, the equivalent circuit model of TENG is a very high voltage source in series with a small capacitor, which is about below nF range^8^,^9^. For the TENG, the power management circuits are worth investigating because TENGs are emerging energy harvesters^10^,^11^. In this case, the most important function of circuit should be improving the charging efficiency since direct integration of TENG with lithium ion batteries will result in huge energy loss due to the ultrahigh TENG impedance^12^.

So far, there are three modes of the TENG: Vertical Contact-Separation mode, In-plane Sliding mode, and Rotation Mode^9^. The first mode is Vertical Contact-Separation: Dielectric-to-Dielectric Case. The output short-circuit current is formed by the two electrodes with the induced charge density^13^. For the mode, the vibration frequency will impact these factors of the velocity, distance and output voltage^14^,^15^. As each contact, the amount of all the electrons and the discharge time are limited, that the current is only about microampere level. For the structure, the average output voltage is about 40 V, and the maximum output voltage is about 125 V, with the average output current is about 0.15 mA, and the maximum output current is 0.3 mA. The maximum output power is about 3 mW^16^. For the Metal-to-Dielectric case, the principle is almost the same as the Dielectric-to-Dielectric case. The maximum output voltage is about 300 V and the output current is low to about 80 μA^17^,^18^. For the useful in-plane sliding mode of the Dielectric-on-Dielectric Case and the Metal-on-Dielectric Case, the maximum output voltage is about 400 V and the output current is about 50 μA^19^,^20^. So the maximum output power is about 8 mW. For the schematic diagram of a cylindrical rotating TENG with 6 strip units, the maximum voltage stays stable about 350 V at sundry rotation speeds. The output current is about 39 μA, when the rotation speed is about 600 r/min^21^,^22^. In the all, the maximum output voltage generated by TENG is about 180 V to 400 V and the output current is about 39 to 175 μA as shown in Table 1. It is very difficult to design circuit for power manager with high efficiency, as the output power is extremely low for the usually energy harvester. The output voltage of TENG is very high, which is easy to destroy some electronic devices, most notably CMOS-integrated circuits and some MOSFET transistors. In this paper, we introduce an interface circuit, using some special devices to solve the high resistance and high voltage with high efficiency to store the power. Making the nano-energy power sources is availability and stability.

In this letter, the lithium ion battery is chosen to store the power of the TENG, whose nominal voltage is 3.6 V^23^. The minimum voltage is about 2.5 V when all the power is consumed. The voltage is decreasing followed by the power consumption. For the lithium ion battery, the lightweight, high voltage, plasticity, and high energy density are its merits. However, the lithium ion battery, without overcharging protection, maybe caused an explosion when is overcharged.

## Results

### Design of the interface circuit

**Figure 1:**
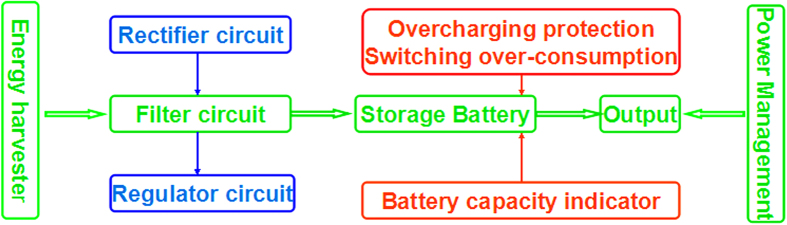
The architecture diagram of the energy harvesting system. The system consists of the energy harvester, storage battery, and power management. The charge circuit involves rectifier, filter, and regulator. The power management includes battery capacity indicator, switch over consumption and overcharging protection.

For the TENG, the output high voltage and low current are not common for the piezoelectric generator^24^,^25^,^26^, solar battery^27^ and wind energy, whose power sources have their own power manger circuits in market, none for the TENG. A novel circuit to manage the power of the TENG is chosen and designed^28^. The buck regulator put the output voltage and current fit to the lithium battery. Protect circuit must be designed, as the over-charge may cause the battery explosive. When the charge battery is consumed, the replace available battery should be considered to extend the battery life. The reliability of the whole system should also be considered as the Fig. 1.

As showing in Fig. 1, energy harvesting system consists of the energy harvester, storage battery and power management. The charge circuit involves rectifier, filter, and regulator. The power management includes battery capacity indicator, switch over consumption, and overcharging protection. The available energy from the environment depends on the time-varying conditions, which is extracted and stored to the battery. Thus, the load can still work, while the environment energy is not available. The buck regulator is used to gather more available energy from harvester to battery. The primary factors of interface circuit include stability, transform efficiency and complexity. With the power management, it must achieve follow function: 1) it can monitor output voltage of the battery and makes sure the voltage is between the Under Voltage (UV) and Over Voltage (OV) threshold to stay in the safe range. 2) It can use LED to monitor the battery capacity.

Considering the reliability, the range of the input voltage should be more free space. Maximum voltage of the TENG is set at 400 V, the lowest current is set at 25 μA and the AC frequency is set among 1 Hz to 100 Hz. Through the interface circuit, the voltage is about 4.2 V, fitting to the lithium ion battery. Before the high voltage and current were delivered to the battery, some transistors with highly breakdown voltage and transformer should be designed to reduce the disturbance of high voltage.

### Rectifier circuit and Filter circuit

**Figure 2:**
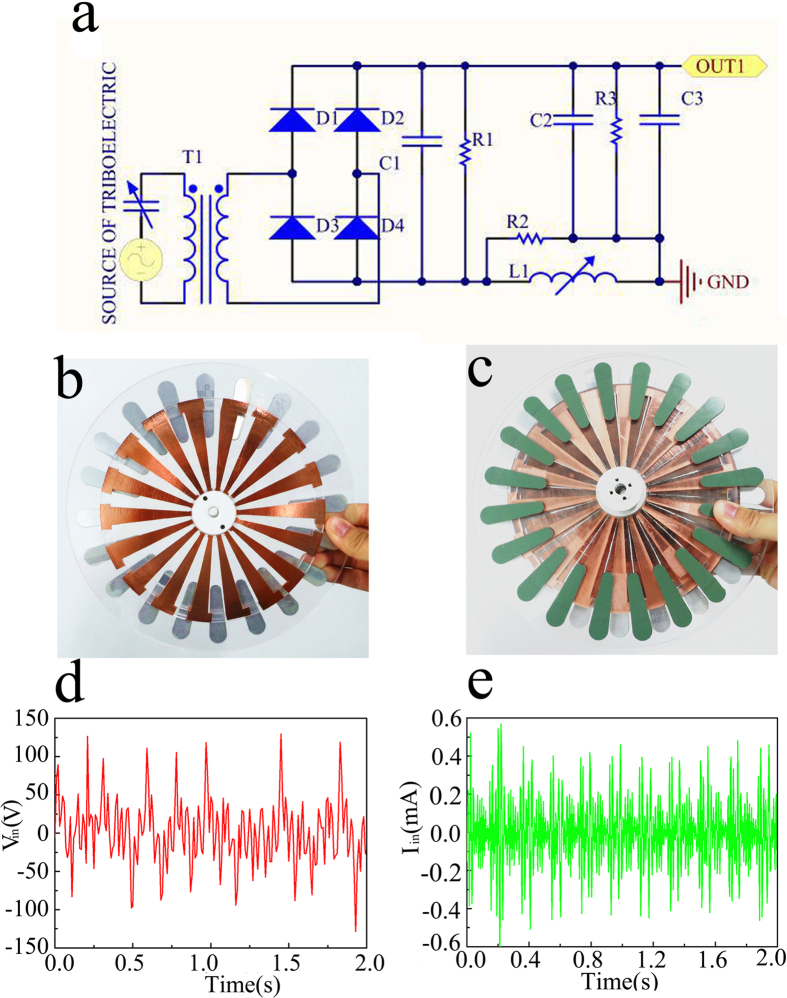
The rectifier and the filter circuit structure and the output voltage and current of the rotating TENG. (**a**) The rectifier and the filter circuit structure. (**b**) Schematic illustration showing a real disk TENG. (**c**) Schematic illustration showing a disk TENG of its back. (**d**) The output voltage. (**e**) The output current.

The equivalent circuit model of TENG should be a voltage source in series with a small capacitor about below nF as the Fig. 2a^8^. Because of the inherent capacitive characteristics, TENGs can only provide unstable AC voltage/current outputs, which is uneasy to simulation the complicate signal^28^. The TENG has a bottom stator and a top rotor, as shown in Fig. 2b,c. On the rotor board, there is a radial array of copper gratings, each of which has a length of 7.2 cm and central angle of 10°. On top of the stator, a radially interdigital structure of the two copper electrodes is covered by a Kapton thin film. Each “finger” of the underneath copper grating also has a central angle of about 10°^14^. The output open voltage and the output short current can be tested to verify the function of the circuit in the Fig. 2d,e. For the output voltage, the form is more liking AC signal, which includes not only one frequency. The Short-circuit output current also exhibits AC behavior, with equal amount of electrons flowing in the opposite directions within one cycle.

**Figure 3:**
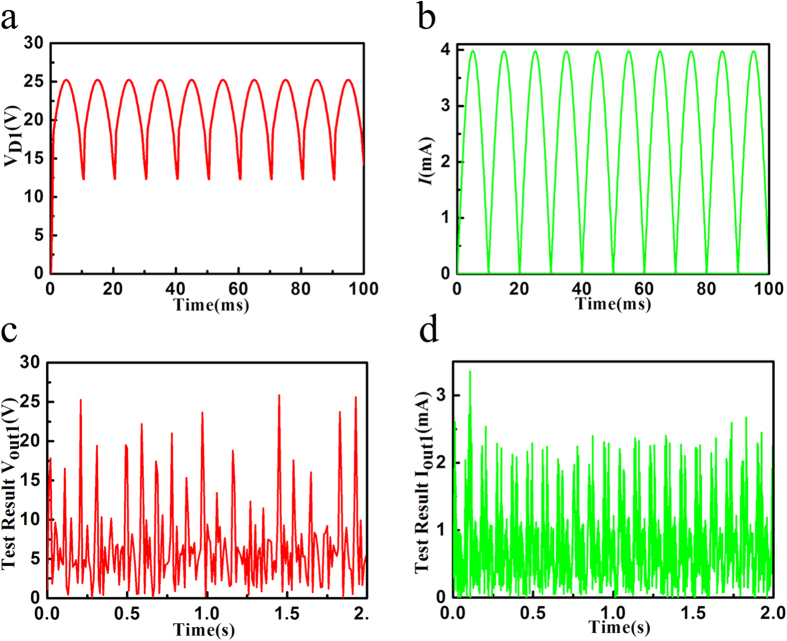
The performance of the Rectifier and Filter circuit. (**a**) The rectified voltage through the transformer and the diodes. (**b**) The rectified current through the transformer and the diodes. (**c**) The test voltage through the transformer and the diodes. (**d**) The test current through the transformer and the diodes.

The rectifier and filter transfer high AC voltage signal into a DC voltage signal, which facilitate the subsequent signal processing. The block diagram of the rectifier and filter is shown in Fig. 2a. The simulation results are shown in the Fig. 3a,b. After rectified, the capacitor can filter some noise. When the capacitance is bigger than 1 uF, it will be the electrolytic capacitor, which has a lot of inductive component. To reduce it, the large electrolytic capacitor is usually in parallel with a small one. In the Fig. 2a, the circuit of π filter consists of C1, C2 and L1 to form a rectified DC voltage. Every capacitor has a resistor in parallel to form the RC loop. The current can be calculated as the equation (1).


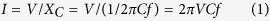


With the voltage of the actually structure tested result is shown in the Fig. 3c, the maximum voltage is about 25 V. The current is about 1 mA. The power is calculated in the equation (2).


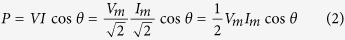


Where *θ* is the angle between the phase of the voltage and the current. The output power can get the maximum when there is no any angle between the phase of the voltage and current.

### Regulator Circuit

**Figure 4:**
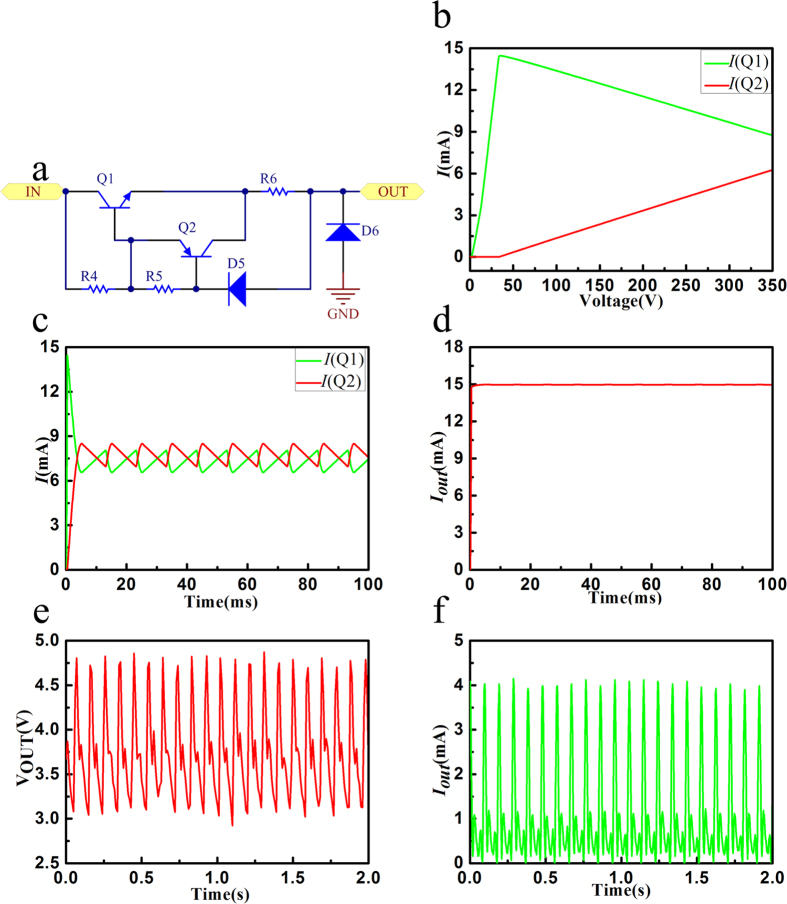
The regulator circuit structure, simulation results, and the test results. (**a**) The regulator circuit. (**b**) The DC Sweep results of the current through the transistor Q1 and Q2. (**c**) The Time Domain results of the current through the transistor Q1 and Q2. (**d**) The Time Domain result of the total output current. (**e**) The actual output voltage of the buck regulator. (**f**) The actual output current of the regulator.

The output voltage of the rectifier and filter is also a challenge for these switch circuits. The highly cost of MOSFET and traditional Pulse Width Modulation(PWM) are limited in the high voltage and low current situation. In this letter, the technology of the StackFET is taken, which uses some economical MOSFET in lower voltage and other modes in the circuit. In the situation, the voltage of widely input range and simple circuit is designed to the regulator circuit. As the Fig. 4a, the two transistors with ultra-low power consumption are used to control and transport the total current, which can charge as much electric as possible into the battery.

The regulator circuit is designed to transfer the output voltage fitting to the lithium ion battery. In the circuit, the zener diode *D*_*5*_ and the base voltage *VB*_*1*_ of the transistor *Q*_*1*_(NPN) are adopted to the reference voltage, as shown in the Fig. 4a. The reference voltage is on the resistor *R*_*6*_, which can get the constant output current. However, as the widely range of the voltage, the current of the zener diode would be varied, and the power would be increasing. The transistor *Q*_*2*_ (PNP) and resistor R_5_ can get the supplementary current. So that the current *I*_*C1*_ is provided on the low input voltage by *Q*_*1*_, and the current *I*_*C2*_ is provided on high input voltage by *Q*_*2*_ in case some interference signal, as shown in the Fig. 4b,c. The total current *I*_*out*_ is offered by *Q*_*1*_ and *Q*_*2*_, which is almost the constant at different input voltage as shown in the equation (3).


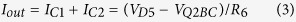


The total current through *R*_*6*_ is about 15 mA, as shown in the Fig. 4d. The constant available voltage can get through the diode D_6_. D_6_ is a transient voltage suppression diode, which connect the power line and grand in parallel. Once there is a transient high voltage, it can release the signal immediately to protect the other components, and it will recover the non-conductive state instantly, which is considered as the reliability design. As the equation (3), V_D5_ − V_Q2BC_ is set 12 V, and V_D5_ is about 15 V. Using D_6_, the reasonable load can be designed to form the available voltage. Through the circuit, it can transport the power of TENG to storing in the battery. Furthermore, The actual voltage and current of the TENG are just like the AC signal shown in the Fig. 2. The average output voltage is about 40 V in the testing TENG. The maximum voltage can get about 125 V. Through the buck regulator, the voltage and current test results are shown in the Fig. 4e,f. The average output voltage is about 4 V. The average current is about 1.12 mA with the load resistance is about 3 kΩ.

### Real-time Monitoring Management

**Figure 5:**
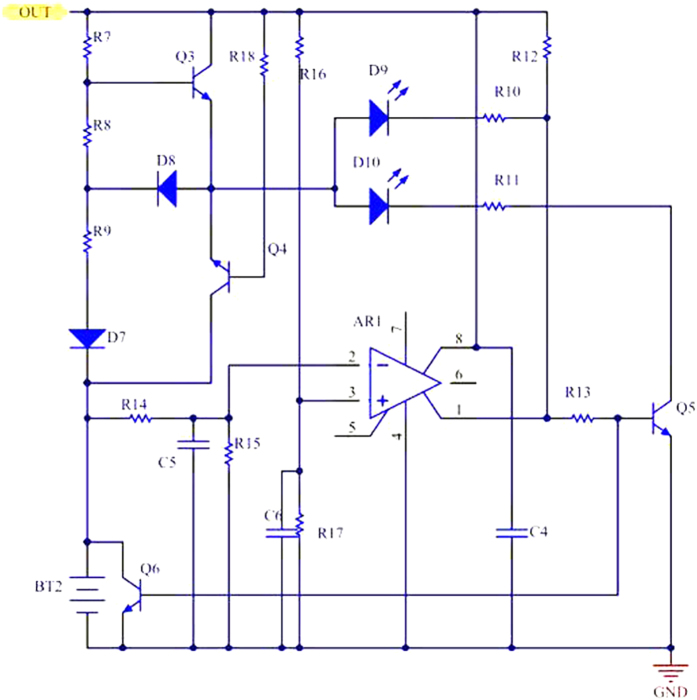
The circuit structures of the battery capacity indicator, the switch over consumption and the overcharging protection. Thinking about the reliability and availability, a real-time monitoring management about the battery is designed.

At the same time, it is very important to design a real-time monitoring management in the battery. The module can monitor its full power or completely consumed. When continuing to charge at the full power situation, it will be exploded^29^. The protect circuit should be designed, and the LED should be lighted to warning. The indicator circuit shows in the Fig. 5a. The voltage of regulators D_6_ is about 5 V. Through the R_16_ and R_17_ the reference voltage is set as 3 V. When the battery gets the full power, its output will be more than the reference voltage and the Operational Amplifier (OA) will output zero. The transistor Q5 and the transistor Q_6_ are off, so that the LED D_9_ will be lighted. Otherwise, the LED D_10_ will be lighted and the battery is power full in the situation. Q_6_ is used to prevent the battery charged again. The capacitor C_4_, C_5_ and C_6_ are mainly to reduce the noise, to be more reliable and stable.

## Discussion

**Figure 6:**
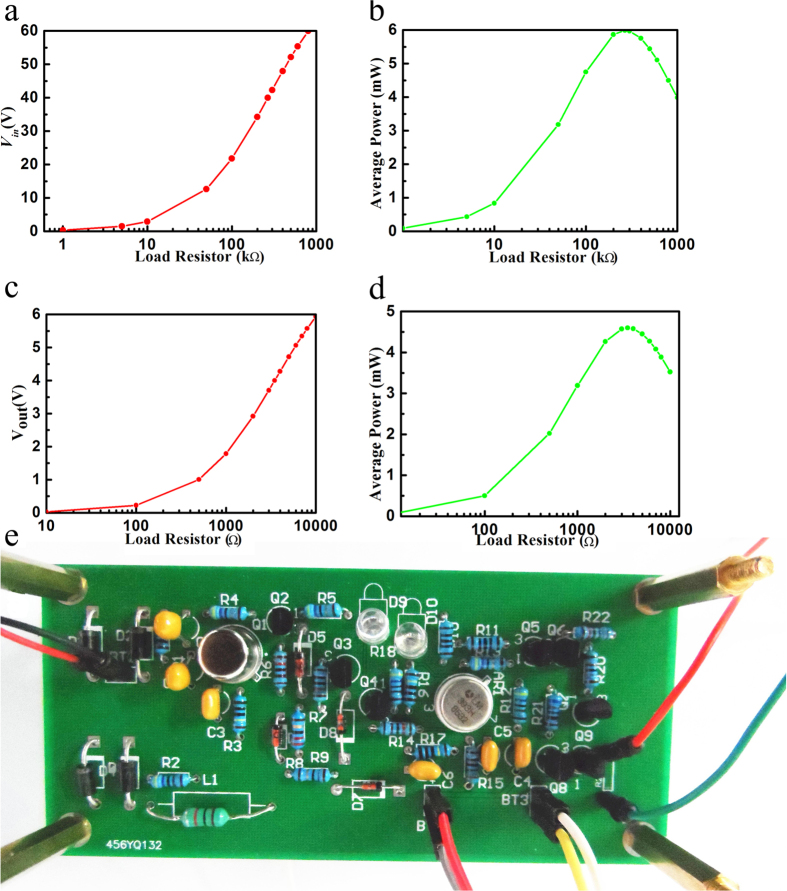
Optimization of the performance of the integrated TENG system and optical photo of the integrated power manger. (**a**) With the integrated TENG system, the DC voltage under different load resistances. (**b**) With the integrated TENG system, average output power under different load resistances. (**c**) With the integrated power manger, the output voltage under different load resistances. (**d**) With the integrated power manger, average output power under different load resistances. (**e**) The actual PCB sample of the system.

By the way, all the power manger system can be fabricated as the sample in the Fig. 6e. The PCB was designed and fabricated to test and verify the performance and efficiency of the power manager system. The test results of the circuit PCB samples is shown in Fig. 6. From the result we can see that as the load resistor is bigger, the voltage would be increase. As the direct of the TENG, when the load is about 260 kΩ, the output maximum power is about 6 mW with the voltage is about 40 V. Through the integrated power manger, when the load is about 3 kΩ, the output maximum power is about 4.5 mW, with the output voltage is about 4 V. The efficiency of the TENG can be calculated as the equation (4).


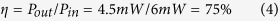


In summary, in the letter for the TENG, the high voltage and low current never appear in the market and research. It is the first time to design the buck regulator for the TENG in this letter. We have mainly solved two problems. Firstly, we have done many researches on the structures and materials of Triboelectric Nanogenerators (TENG) to explain the phenomenon that the maximum voltage is stable and the current is increasing. The output voltage of the TENG is high about 100–400 V, and the output current is small about 25 μA, which is not easy to use in the electronic devices. Secondly, a novel interface circuit with the high-voltage buck regulator for TENG is introduced firstly in this manuscript. The interface circuit can transfer the output signal of the TENG into the signal fit to a lithium ion battery. Further, the reliability and availability for the lithium ion battery and the circuit are discussed. The interface circuit is simulated using the Cadence software and verified through PCB experiment. The buck regulator can achieve 75% efficiency for the High-Voltage TENG. In that situation, the power of the TENG can drive some small electronics^30^,^31^, which could be used widely for the remote and mobile environmental sensors, homeland security, and even wearable personal electronics^32^,^33^.

## Methods

### Design of the interface circuit

As we use the the Triboelectric Nanogenerators (TENG) from Dr. Zhang Chi, the Maximum voltage of the TENG is set at 400 V, the lowest current is set at 25 μA and the AC frequency is set among 1 Hz to 100 Hz. The energy harvesting system consists of the energy harvester, storage battery and power management. The charge circuit involves rectifier, filter, and regulator. The power management includes battery capacity indicator, switch over consumption, and overcharging protection to ensure the harvesting system work.

### Measurement of the TENG

To measure the triboelectric output voltage, a 100× probe (HP9258) was connected to the oscilloscope (Agilent DSO-X 2014A). The equivalent internal resistance is 260 kΩ and the corresponding settings in the oscilloscope was adjusted to 100×. To measure the triboelectric output current, a 260 kΩ external resistance was connected parallel to the TENG and the measured signal was calculated to the current value. In the electromagnetic part, 260 kΩ probe was used to measure the voltage and 100 Ω external resistance was parallel connected to measure the current.

### Simulink circuit

The Simulink circuit includes three parts: Rectifier circuit and Filter circuit, Regulator Circuit, and Real-time Monitoring Management. These are simulated using the Cadence software and verified through PCB experiment. The test result can be shown in the correspondence figure, and the PCB is showing in the Fig. 6e.

## Additional Information

**How to cite this article**: Luo, L.-C. *et al.* A Low Input Current and Wide Conversion Ratio Buck Regulator with 75% Efficiency for High-Voltage Triboelectric Nanogenerators. *Sci. Rep.* **6**, 19246; doi: 10.1038/srep19246 (2016).

## Figures and Tables

**Table 1:**
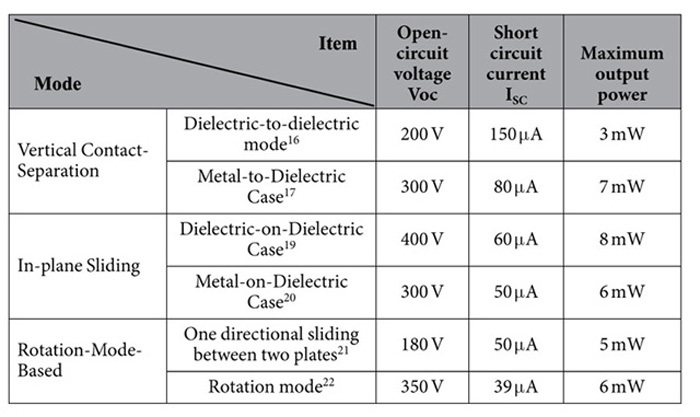
The parameters of the typical TENG.
